# Maternal Complications and Hemodynamic Changes Following Intrauterine Interventions for Twin-to-Twin Transfusion Syndrome in Monochorionic Diamniotic Twin Pregnancies

**DOI:** 10.3390/jcm8050605

**Published:** 2019-05-02

**Authors:** Patrick Greimel, Angela Zenz, Bence Csapó, Martin Haeusler, Uwe Lang, Philipp Klaritsch

**Affiliations:** Division of Obstetrics and Maternal Fetal Medicine, Department of Obstetrics and Gynecology, Medical University of Graz, Auenbruggerplatz 14, 8036 Graz, Austria; patrick.greimel@medunigraz.at (P.G.); angela.zenz@hotmail.com (A.Z.); bence.csapo@medunigraz.at (B.C.); martin.haeusler@medunigraz.at (M.H.); Uwe.Lang@klinikum-graz.at (U.L.)

**Keywords:** monochorionic diamniotic twins (MCDA), twin-to-twin transfusion syndrome (TTTS), fetoscopy, amniodrainage, maternal complications, mirror syndrome, hemodilution

## Abstract

Twin-to-twin transfusion syndrome (TTTS) is a challenging complication in monochorionic diamniotic (MCDA) twins. Intrauterine interventions, such as fetoscopic laser ablation and cord occlusion followed by amniodrainage, are established treatments. Little is known about maternal complications and hemodynamics following these interventions. We performed a retrospective analysis of maternal procedure-related complications and the impact of such procedures on maternal hemodynamics and blood characteristics. Within the study period, 100 women with severe TTTS treated by fetoscopic laser ablation (FLA) or cord occlusion (CO) were identified. Clinically relevant maternal complications were reported in four (4%) cases. There was a significant decrease in hemoglobin, hematocrit, and albumin between admission and postoperative measurements (all *p* < 0.001). Systolic and diastolic blood pressure, as well as maternal heart rate, decreased from time of skin suture to postoperative measurements (all *p* < 0.001). Within a 24 h interval, there was a positive correlation between hematocrit (Spearman’s rho 0.325; *p* = 0.003), hemoglobin (Spearman’s rho 0.379; *p* < 0.001), and albumin (Spearman’s rho 0.360; *p* = 0.027), and the amount of amniodrainage during the intervention. Maternal procedure-related complications are relatively rare. Significant hemodynamic alterations and maternal hemodilution are common clinical findings following intrauterine interventions.

## 1. Introduction

Twin-to-twin transfusion syndrome (TTTS) complicates about 10% of monochorionic diamniotic twin pregnancies [1,2]. The condition is caused by unbalanced intertwin blood flow through vascular anastomoses on the placental surface, leading to volume depletion of the donor twin and volume overload of the recipient twin [3,4,5,6]. Fetoscopic laser ablation (FLA) of placental anastomoses is currently the best available treatment for TTTS, yielding double survival rates of more than 60% in large recent series [7,8,9,10,11]. In cases with unfavorable prognosis or technical issues hampering successful FLA, selective feticide by cord occlusion (CO) may be offered as an alternative approach [12,13]. All of these interventions are usually completed by amniodrainage of the recipient’s excess fluid to achieve normal amniotic fluid levels and thereby decrease intrauterine pressure.

While the majority of research is focusing on advances in fetal treatment and long-term outcome, little is known about the mother’s clinical and circulatory response following intrauterine procedures in cases of TTTS. There is only a limited number of reports on maternal surgery-related complications. Significant differences in surgical complication rates between structured and unstructured adverse outcome assessment were found. The overall rate of maternal adverse events was found to be 17%, while intermediate to severe problems occurred in about 4% [14]. Still, FLA procedures are considered relatively safe for pregnant women, especially when performed in high-volume centers [15,16]. However, surgery-related complications are not the only concern in women treated for TTTS. Maternal circulation has to tolerate significant adaptational changes even during uncomplicated singleton pregnancy [17]. In twin pregnancies, and especially in those complicated by TTTS, challenges in terms of mechanical and cardiocirculatory stress affect women to a much higher degree [18,19]. In 2000, De Lia et al. described signs suggestive of maternal hemodilution in 109 cases of mid-trimester TTTS following FLA and amniodrainage. This was the first report suggesting that hemodynamic changes following such interventions may also have an impact on maternal circulation [20]. Later, the amount of amniotic fluid drained was linked to the degree of changes between pre- and postoperative hematocrit and hemoglobin levels [21]. It seems that the maternal cardiocirculatory system is stressed by high-volume amniodrainage in particular, leading to a decrease in mean arterial blood pressure and total vascular resistance, and an increase in cardiac output and stroke volume. These hemodynamic alterations were pronounced within the first six postoperative hours and persisted at least 24 h after treatment [22].

We sought to investigate maternal procedure-related complications following intrauterine interventions for TTTS as well as the impact of such procedures on maternal hemodynamics.

## 2. Materials and Methods

In this retrospective single-center cohort study, all women affected by TTTS who were treated at the Department of Obstetrics and Gynecology at the Medical University of Graz, Austria, between August 2010 and January 2018 were included. Data were derived from the local registry on monochorionic pregnancies (“MonoReg”). This registry study was approved by the ethics committee of the Medical University of Graz (29-105 ex 16/17). Additional data were collected via electronic databases Medocs© (SAP, Walldorf, Germany) and PIA© (ViewPoint, GE Healthcare, Solingen, Germany) as well as written medical records. Data were processed in Microsoft Excel© files (Microsoft Corporation, Redmond, WA, USA).

The current study was approved by the Ethics Committee of the Medical University of Graz, Austria, registered at Institutional Review Board Registry as IRB00002556 (29-215 ex 16/17). All patient data were pseudonymized. Only authorized persons had access to original datasets.

### 2.1. Study Population

Data of all women with monochorionic diamniotic twin pregnancies treated by FLA or CO in the case of severe TTTS were selected, while those who underwent intrauterine therapy for indications other than advanced TTTS were excluded from the analyses. At our institution, intrauterine treatment (i.e., FLA or CO) is generally offered to all cases with Quintero stage I or higher, as defined by polyhydramnios in the recipient’s sac (i.e., deepest vertical pocket (DVP) measuring more than 8 cm before 20 weeks of gestation and more than 10 cm beyond 20 weeks of gestation) in combination with oligohydramnios in the donor’s sac (i.e., DVP of less than 2 cm) [8,23].

### 2.2. Surgical Procedures

Under local anesthesia (10 mL of 0.5 mg mepivacaine) and continuous intravenous administration of remifentanil (0.1–0.2 µg/kg/min), a 10 Fr cannula (Cook Medical, Bloomington, IN, USA) loaded with a reusable trocar (Storz, Tuttlingen, Germany) was percutaneously inserted into the amniotic cavity under ultrasound guidance, as previously described [24,25]. A 3.3 mm fetoscope (model number 11506 AA or 11508 AA, Karl Storz, Tuttlingen, Germany) was introduced, and selective laser ablation of intertwin anastomoses was performed by using a diode laser (Medilas D Multibeam; Dornier MedTech, Weßling, Germany) via 0.4 mm laser fiber with 10–25 W. Subsequently, laser coagulation of the entire vascular equator (Solomon technique) was performed whenever technically feasible [26]. For selective feticide, a bipolar forceps (Everest MOLly Forceps, Gyrus ACMI, Maple Grove, MN, USA) was inserted under sonographic view and bipolar coagulation of the umbilical cord was performed with 25–30 W at up to three adjacent sections of the cord, until arrest of blood flow was confirmed by Doppler ultrasound [12,25]. All interventions were completed by amniodrainage of the recipient’s amniotic fluid until a DVP of about 6 cm was reached. Amniodrainage was carried out via the 10 Fr cannula and the drained volume was measured using a graduated beaker. The procedure was constantly monitored by ultrasonography. A Lyostypt© collagen plug (Braun, Melsungen, Germany) was placed at the insertion site. Intracutaneous skin closure was accomplished by a single suture of Monocryl© (Ethicon, Somerville, MA, USA). A urinary catheter was placed at surgery for 24 h and production of urine was monitored. Perioperative intravenous antibiotic prophylaxis (cefazolin) was administered for 24 h. Tocolysis was dependent on gestational age at surgery: Before 20 weeks of gestation, oral tocolysis with 20 mg nifedipine was administered twice daily for 48 h, while in the case of clinically apparent uterine contractions beyond 20 weeks atosiban (1.5 µg/kg/min) was given intravenously for 48 h following the intervention. In general, there was no relevant perioperative fluid administration. From two hours after the intervention, women were allowed unrestricted fluid and food intake.

### 2.3. Outcome Parameters

Surgical complications, perinatal survival, and maternal parameters including maternal age, gestational age at intervention, maternal body mass index (BMI), and Quintero stage at intervention were documented. Total volume of amniodrainage was recorded. Maternal serum hemoglobin (Hb), hematocrit (Hct), and albumin were measured at admission (Tadm), 2 h (T1), and 24 h (T2) after the intervention. Systolic and diastolic arterial blood pressures were measured at the time of skin suture (Tsut), as well as 2 h (T1) and 24 h (T2) after surgery. Correlation analysis of amniodrainage volume and postoperative levels of Hb, Hct, and albumin, as well as systolic and diastolic blood pressure, were conducted.

### 2.4. Statistics

Data were tested for normal distribution using the Kolmogorov–Smirnov and Shapiro–Wilk tests. Correlation analyses between related parameters were performed with Spearman´s rho if a dataset was not normally distributed. Not-related parameters were tested with the Wilcoxon test. In the case of normal distribution, Student’s *t*-test was performed. A probability of *p* < 0.05 was considered statistically significant. Analyses were carried out using SPSS Statistics 23 (IBM, Armonk, NY, USA).

## 3. Results

Within the study period, 100 women with severe TTTS treated by FLA or CO were identified. Maternal age at intervention was 34.5 years (25–49) and maternal BMI was 24.55 (17.85–40.53). Gestational age at intervention was 19.86 (16–28) weeks (see Table 1). The majority of cases (79%) were classified as Quintero stage II and III, respectively [23].

Seventy-five (75%) FLA procedures were satisfactorily feasible from a technical point of view. In six (6%) cases, FLA could not be performed along the whole equator and FLA was therefore considered as being incomplete during surgery. In three (3%) of these cases, CO was performed before amniodrainage, while the other three (3%) cases were treated by amniodrainage alone. In 19 (19%) cases, CO was performed as a primary approach. The median volume of amniodrainage was 1420 mL (110–4040) (see Table 2). There was no statistically significant difference between the volume of amniodrainage after CO and FLA (*p* = 0.126). Median amniodrainage volume after CO was 1010 mL (110–3340) while median amniodrainage volume following FLA was 1440 (140–4040).

### 3.1. Maternal Procedure-Related Complications

Clinically relevant maternal complications were reported in four (4%) cases. There was one (1%) case of retroplacental hematoma, and three (3%) cases of “mirror syndrome”. Detailed description of clinical features is given in Table 3. The rate of iatrogenic preterm premature rupture of the membranes (pPROM) within two weeks after the intervention was 5%. Single intrauterine fetal demise (sIUFD) and double intrauterine fetal demise (dIUFD) after FLA occurred in 14% and 3%, respectively. There were four (4%) cases of premature delivery before 34 weeks of gestation, two (2%) cases of pregnancy loss before viability, and one (1%) case complicated by cervical insufficiency within two weeks after the intervention (see Table 4). 

### 3.2. Maternal Serum Parameters and Hemodynamics

**Figure 1:**
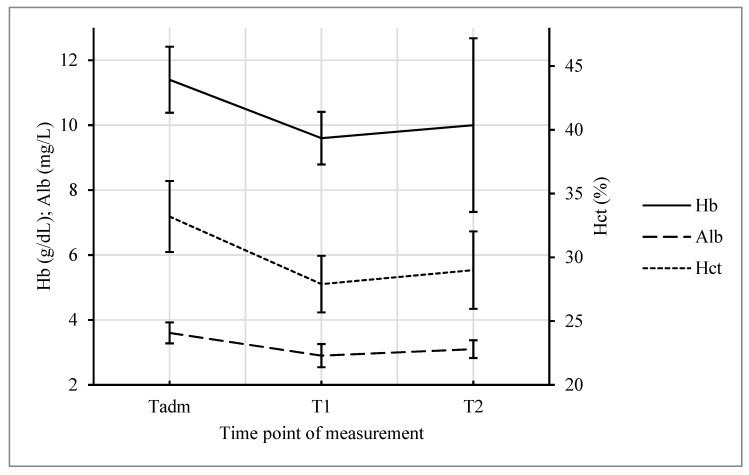
Hemoglobin (Hb), hematocrit (Hct), and albumin (Alb) before admission (T_adm_), 2 h (T1), and 24 h (T2) after the intervention, respectively. Data presented as mean values (SD) (T_adm_ to T1 all *p* < 0.001).

**Figure 2:**
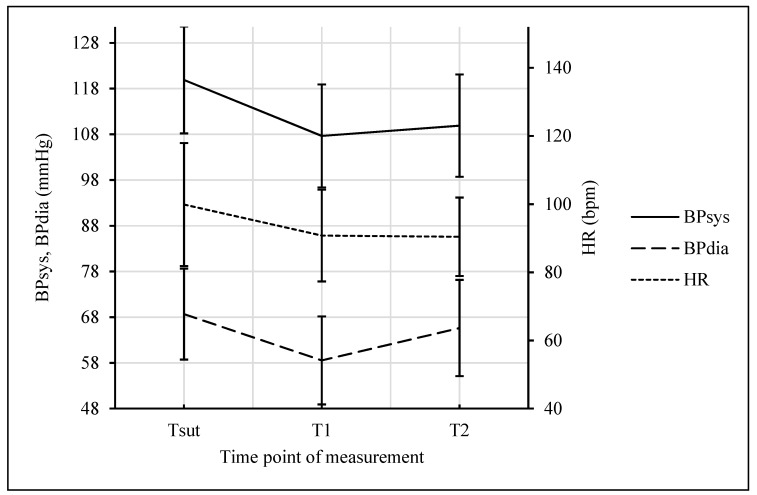
Systolic (BPsys), diastolic blood pressure (BPdia), and maternal heart rate (HR) at skin suture (T_sut_), 2 h (T1), and 24 h (T2) after the intervention, respectively. Data presented as mean values (SD) (T_sut_ to T1 all *p* < 0.001).

There was a significant decrease in Hb, Hct, and albumin between Tadm and T1 (all *p* < 0.001). Mean Hb dropped from 11.60 ± 1.00 to 9.61 ± 0.80 g/dL, mean Hct from 33.56% ± 2.84% to 27.78% ± 2.23%, and albumin from 3.65 ± 0.36 to 2.82 ± 0.36 mg/L, respectively. From T1 to T2, variables did not change significantly (see Figure 1). Systolic (BPsys) and diastolic blood pressure (BPdia), as well as maternal heart rate (HR), decreased from Tsut to T1 significantly (all *p* < 0.001). Mean BPsys dropped from 120.13 ± 11.10 to 107.80 ± 11.11 mmHg, mean BPdia from 69.15 ± 9.84 to 58.31 ± 9.45 mmHg, and mean HR from 99.92 ± 18.51 to 90.70 ± 13.33 bpm, respectively. Mean BPdia increased significantly from 58.32 ± 9.45 at T1 to 65.88 ± 10.63 mmHg at T2, while BPsys showed the same trend from 107.51 ± 11.27 at T1 to 109.67 ± 10.90 mmHg at T2, but was not statistically significant. Maternal HR was stable from T1 to T2 (see Figure 2).

**Figure 3:**
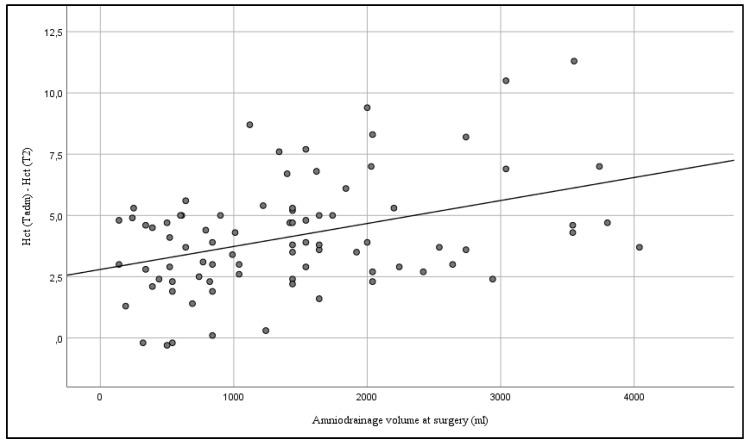
Correlation between the volume of amniodrainage at surgery and changes of maternal serum hematocrit (Hct) within 24 h after the intervention (Spearman’s rho 0.325; *p* = 0.003).

**Figure 4:**
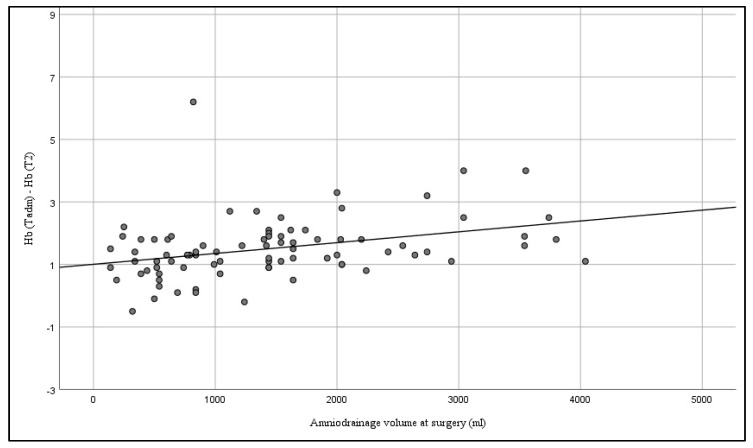
Correlation between the volume of amniodrainage at surgery and changes of maternal serum hemoglobin (Hb) within 24 h after the intervention (Spearman’s rho 0.379; *p* < 0.001).

**Figure 5:**
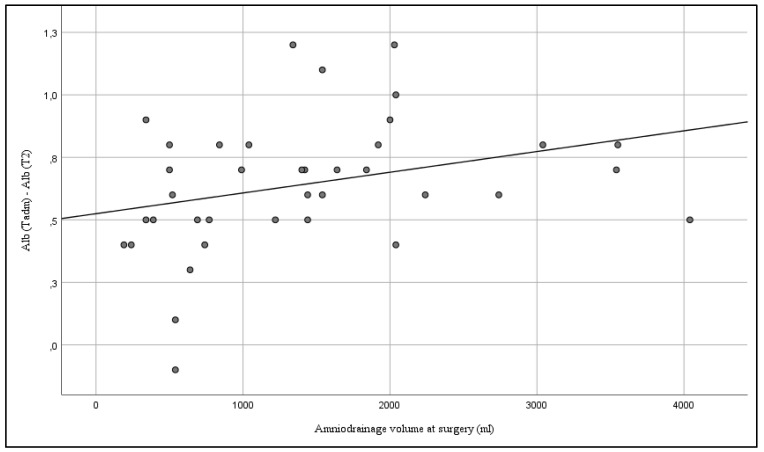
Correlation between the volume of amniodrainage at surgery and changes of maternal serum albumin (Alb) within 24 h after the intervention (Spearman’s rho 0.360; *p* = 0.027).

There was a significant correlation between the volume of amniodrainage and the effects on maternal blood characteristics. Within a 24 h interval, there was a positive correlation between Hct (Spearman’s rho 0.325; *p* = 0.003) (see Figure 3), Hb (Spearman’s rho 0.379; *p* < 0.001) (see Figure 4), and albumin (Spearman’s rho 0.360; *p* = 0.027) (see Figure 5), and the amount of amniodrainage during the intervention.

There was a significant correlation between maternal BMI and the effect on maternal hemodynamics. Within a 24 h interval, there was a negative correlation between maternal BMI and systolic blood pressure (Spearman’s rho −0.213; *p* = 0.044). Correlation analysis between maternal BMI and changes of hematocrit or between BMI and surgical complications showed no significant results. Correlation analysis between amniodrainage volume and changes in postoperative diuresis as well as changes in serum sodium within 24 h was not significant.

High-volume amniodrainage at surgery was a significant intraoperative marker predictive for severe postoperative maternal hemodilution, defined as Hb ≤ 8.5 g/dL and Hct ≤ 25%. This group showed a median drainage volume of 2440 mL (240–3800), while the control group had a median drainage volume of 1240 mL (110–4040) (*p* = 0.004).

## 4. Discussion

Severe maternal postoperative complications attributable to intrauterine interventions were relatively rare with an incidence of 4%. This seems to be comparable with previously reported data on severe maternal complications with a frequency of around 1%–6% [14,15]. Interestingly, serious maternal hemodynamic alterations, usually referred to as mirror syndrome or Ballantyne´s syndrome, occurred quite often (3%). First described by Ballantyne in 1892, this syndrome summarizes diverse immunological and non-immunological fetal conditions which lead to subsequent fetal and placental hydrops and a concomitant “maternal dropsy”, anemia, hypoproteinemia, albuminuria, and oliguria [27]. The underlying pathophysiology is still poorly understood. We report on three cases consistent with the features of Ballantyne’s syndrome in our study population. In case A, the donor twin showed acute placental hydrops due to anemia requiring intrauterine blood transfusion during fetoscopy. Maternal hemodilution with the lowest hemoglobin of 8.9 g/dL was found at 2 h postoperatively. The mother presented clinical signs indicating volume overload and increased right ventricular load. Both fetuses died after the intervention. Case B had a fulminant course with rapidly progressive diseases following the laser intervention with dyspnea, hypotension, tachycardia, and significant hemoglobin drop (lowest hemoglobin 7.2 g/dL at 18 h postoperatively). The recipient twin, who had suffered from severe cardiac compromise before FLA, demised shortly after the procedure. Clinical signs of mirror syndrome were falsely interpreted as severe intraabdominal bleeding by the staff on duty, and were therefore treated with a massive transfusion of red blood cell concentrates (1265 mL) and isotonic fluid administration (3500 mL), thus aggravating fluid overload to bilateral lung edema. During this period, the donor twin died as well, most likely due to severe maternal hypotension. Despite double fetal demise, emergency cesarean section was performed for maternal indication. There were no signs of significant intraabdominal blood loss. The patient was transferred to the intensive care unit where parenteral diuretics and albumin were given to restore volume homeostasis, leading to complete remission of clinical problems within 3 days. In case C, the surviving twin showed transient placental hydrops after CO, and had spontaneous remission 2 days after the intervention. The mother showed severe anemia with hemoglobin of 7.8 g/dL 3 h postoperatively, however, there were no clinical signs of maternal hemorrhage. Similarly, these cases showed amniodrainage volumes and gestational weeks above average. This might be a clue to underlying mechanisms, given that amniodrainage volume seems to be important for changes in maternal blood characteristics and hemodynamics after intrauterine interventions, as previously reported [21,22].

The rate of iatrogenic pPROM was as low as 5% within 2 weeks after the intervention. Compared to Yamamoto et al. who reported a rate of 12% within 3 weeks after FLA [16], and Rustico et al. who reported a rate of 9% within one week [15], we found pPROM to be slightly lower in our study population than was previously reported. The routine usage of a collagen plug for the closure of the site of trocar insertion might be an explanation, although recent studies did not confirm a benefit of this technique [28,29].

Our data support findings by Nizard and co-workers on maternal hemodynamic effects after intrauterine intervention and subsequent amniodrainage [22]. We demonstrated a significant drop of mean systolic and diastolic blood pressure and maternal heart rate from skin suture to 2 h postoperatively in all patients irrespective of the drained volume. Nizard et al. showed significant differences between two groups: one with an amniodrainage volume above 1000 mL and hemodynamic alterations (group A), and another group with a drainage volume below 1000 mL and absent signs of maternal effects (group B). We assume that maternal hemodynamic effects may have been detectable in our entire study population because the median drainage volume corresponded to group A in Nizard’s study. Due to the retrospective design of our study, it was not possible to evaluate additional maternal hemodynamic parameters.

Furthermore, our data confirm previous studies on changes in maternal blood characteristics following high-volume amniodrainage [20,21,22]. De Lia mentioned poor maternal nutritional status linked to hyperemesis in early pregnancy as an underlying factor for the findings of anemia and hypoproteinemia. He proposed a net efflux of fluid from the mother to the recipient twin due to higher colloid osmotic pressure in the recipient twin, thus aggravating TTTS. We suppose that malnutrition was not an issue in our study population as our population presented with a mean BMI of 25.17 and preoperative blood characteristics comparable to a historic population of healthy controls cited by De Lia et al. [20]. We interpret maternal hypoproteinemia and anemia as being postoperative alterations in the maternal circulatory system caused by amniodrainage and not by preexisting malnutrition. A striking argument for this being an intervention-induced phenomenon is the rapid development after surgery and the obvious association to higher volumes of amniodrainage as demonstrated in a correlation analysis described above and also observed by Huber et al. [21]. We strongly support the hypothesis of maternal hemodilution due to amniodrainage. Signs of mild to moderate hemodilution in nearly every patient after high-volume amniodrainage could be an attenuated variant of Ballantyne’s syndrome. The pathophysiological circumstances are still unclear, but the placenta might play an important role during decompression of the uterine and abdominal compartment.

Patients characteristics were comparable with previous reports on maternal complications after FLA [15,16,22]. Certainly, the limitations of having a retrospective study design apply to our study as well. The strength of this study, however, is the structured approach in documenting and reporting maternal postoperative problems.

Presented data on maternal risks may be used by clinicians for preoperative counseling and postoperative interpretation of medical conditions and laboratory results. Surgeons should avoid misinterpretation of maternal blood characteristics such as decreasing Hb and Hct as acute maternal hemorrhage, since postoperative hemodilution seems to be the underlying mechanism in most cases.

## Figures and Tables

**Table 1:** Patient characteristics and Quintero stage at intervention.

*N* *	100 (100)
Maternal age, years ^§^	34.50 (25–49)
Gestational age at intervention, weeks ^§^	19.86 (16–28)
BMI ^§^	24.55 (17.85–40.53)
Quintero stage at intervention *	
Stage I	17 (17%)
Stage II	33 (33%)
Stage III	46 (46%)
Stage IV	3 (3%)
Stage V	1 (1%)

* Data are presented as number (%); ^§^ Data are presented as median (range). Abbreviations: BMI body mass index.

**Table 2:** Type of intervention and volume of amniodrainage at intervention.

Type of Intervention	*N* *
CO + AD	19 (19)
FLA + AD	75 (75)
Incomplete FLA + AD	3 (3)
Incomplete FLA + CO + AD	3 (3)
Total number of interventions	100 (100)
Volume of amniodrainage mL ^§^	1420 (110–4040)

* Data are presented as number (%); ^§^ Data are presented as median (range). Abbreviations: CO cord occlusion; AD amniodrainage; FLA fetoscopic laser ablation.

**Table 3:** Maternal complications related to intrauterine surgery within 2 weeks after the intervention.

Maternal Complications within 2 Weeks after Intervention	*N* *	Case Reference	GA at Intervention (Weeks)	Quintero Stage at Intervention	Type of Intervention	AD Volume (mL)	Maternal Hemodilution	Cardiac Decompensation	Clinical Signs of Maternal Hemorrhage	Lowest Hemoglobin (g/dL)	ICU Admission	Fetal Outcome
Mirror syndrome	3 (3)	A	25 + 2	II	FLA; IUTx	2000	yes	yes	no	8.9	no	dIUFD
		B	26 + 1	III	FLA	2500	yes	yes	no	7.2	yes	dIUFD
		C	19 + 6	II	CO	2600	yes	yes	no	7.8	no	Single survival
Retroplacental hematoma	1 (1)	D	19 + 6	III	CO	750	no	no	no	8.6	no	Single survival
Wound problems	0											
Intraabdominal bleeding	0											
Chorioamnionitis	0											
Amniotic fluid leakage into the peritoneal cavity	0											
No complication	96 (96)											
Total maternal complications	4 (4)											

* Data are presented as number (%). Abbreviations: GA, gestational age; CO, cord occlusion; AD, amniodrainage; FLA, fetoscopic laser ablation; IUTx, intrauterine blood transfusion; dIUFD, double intrauterine fetal demise; ICU, Intensive care unit.

**Table 4:** Obstetric and fetal complications within 2 weeks after the intervention.

Type of Complication	*N* *
No complication	74/100 (74)
Double survival after FLA	65/78 (83)
sIUFD after FLA	11/78 (14)
dIUFD after FLA	2/78 (3)
Survival following CO	21/22 (95)
pPROM	5/100 (5)
Abortion	2/100 (2)
Intrauterine transfusion (IUTx)	1/100 (1)
Premature labor <34 weeks	4/100 (4)
Cervical insufficiency	1/100 (1)
Total complications	26/100 (26)

* Data are presented in number per specific intervention (%) or number per all interventions (%), respectively. Abbreviations: FLA fetoscopic laser ablation; sIUFD single intrauterine fetal demise; dIUFD double intrauterine fetal demise; CO cord occlusion; pPROM preterm premature rupture of membranes.
